# A Recurrent Stop-Codon Mutation in Succinate Dehydrogenase Subunit B Gene in Normal Peripheral Blood and Childhood T-Cell Acute Leukemia

**DOI:** 10.1371/journal.pone.0000436

**Published:** 2007-05-09

**Authors:** Bora E. Baysal

**Affiliations:** 1 Department of Obstetrics, Gynecology and Reproductive Sciences, University of Pittsburgh School of Medicine, University of Pittsburgh, Pittsburgh, Pennsylvania, United States of America; 2 Department of Human Genetics, Graduate School of Public Health, University of Pittsburgh, Pittsburgh, Pennsylvania, United States of America; Lehigh University, United States of America

## Abstract

**Background:**

Somatic cytidine mutations in normal mammalian nuclear genes occur during antibody diversification in B lymphocytes and generate an isoform of apolipoprotein B in intestinal cells by RNA editing. Here, I describe that succinate dehydrogenase (SDH; mitochondrial complex II) subunit B gene (*SDHB*) is somatically mutated at a cytidine residue in normal peripheral blood mononuclear cells (PBMCs) and T-cell acute leukemia. Germ line mutations in the *SDHB*, *SDHC* or *SDHD* genes cause hereditary paraganglioma (PGL) tumors which show constitutive activation of homeostatic mechanisms induced by oxygen deprivation (hypoxia).

**Principal Findings:**

To determine the prevalence of a mutation identified in the *SDHB* mRNA, 180 samples are tested. An *SDHB* stop-codon mutation c.136C>T (R46X) is present in a significant fraction (average = 5.8%, range = less than 1 to 30%, n = 52) of the mRNAs obtained from PBMCs. In contrast, the R46X mutation is present in the genomic DNA of PBMCs at very low levels. Examination of the PBMC cell-type subsets identifies monocytes and natural killer (NK) cells as primary sources of the mutant transcript, although lesser contributions also come from B and T lymphocytes. Transcript sequence analyses in leukemic cell lines derived from monocyte, NK, T and B cells indicate that the mutational mechanism targeting *SDHB* is operational in T-cell acute leukemia. Accordingly, substantial levels (more than 3%) of the mutant *SDHB* transcripts are detected in five of 20 primary childhood T-cell acute lymphoblastic leukemia (T-ALL) bone marrow samples, but in none of 20 B-ALL samples. In addition, distinct heterozygous *SDHB* missense DNA mutations are identified in Jurkat and TALL-104 cell lines which are derived from T-ALLs.

**Conclusions:**

The identification of a recurrent, inactivating stop-codon mutation in the *SDHB* gene in normal blood cells suggests that *SDHB* is targeted by a cytidine deaminase enzyme. The *SDHB* mutations in normal PBMCs and leukemic T cells might play a role in cellular pre-adaptation to hypoxia.

## Introduction

Oxygen deprivation (hypoxia) induces adaptive responses in most organisms and cell types [1] and may contribute to pathogeneses of common human diseases. Several adaptive responses to hypoxia are mimicked by hereditary paraganglioma (PGL), a human genetic disorder characterized by the development of tumors from the hypoxia-sensitive paraganglionic tissues and caused by germ line heterozygous inactivating mutations in the nuclear-encoded *SDHB*, *SDHC* or *SDHD* subunit genes of mitochondrial complex II (succinate dehydrogenase; SDH; succinate-ubiquinone oxidoreductase) [2], [3]. PGL mutations or chronic hypoxic exposure predispose to carotid body (CB) paragangliomas [2]. The PGL tumors are highly vascular and show global gene expression profiles of angiogenesis and hypoxic-pathway activation [4]. Lower altitudes are associated with decreased penetrance/expressivity and increased prevalence of *SDHD* mutations [5]. Heterozygous inactivation of the mouse *Sdhd* gene leads to persistent hypoxic stimulation of the CB paraganglionic cells [6]. These data strongly suggest that constitutive activation of hypoxia-sensing and signaling pathways, presumably as a consequence of accumulation of reactive oxygen species [7], succinate [8] and/or another messenger molecule, underlies PGL pathogenesis. Whether PGL gene mutations predispose to non-paraganglionic tumors or system abnormalities is a subject of ongoing investigations [8], [9].

In contrast to *SDHB* and *SDHC*, mutations in *SDHD* display a transmission pattern of maternal genomic imprinting (inactivation). *SDHD* mutations cause PGL type 1 (PGL1) after a paternal but not a maternal transmission. Although no maternal transmission of PGL1 has ever been documented, its molecular basis remains unclear. Non-paraganglionic tissues display bi-allelic expression of *SDHD*, suggesting that imprinting may be confined to paraganglia [2]. In this report, I demonstrate that the *SDHB* subunit gene is targeted by a somatic mutational mechanism in normal peripheral blood and childhood T-cell acute leukemia.

## Methods

### Samples

Peripheral bloods (10–30 mL) were obtained by venipuncture into EDTA-containing tubes from consenting adults of both sexes and shipped to author's laboratory at room temperature. Peripheral blood mononuclear cells (PBMCs) were isolated by centrifugation using BD vacutainer CPT™ tubes (Beckton-Dickinson) and stored at −135°C or in liquid nitrogen in freezing media, which was composed of 90% growth media containing RPMI-1640 (CellGro) and 15% fetal bovine serum (CellGro) and 10% DMSO (Sigma). A subset of PBMCs was used to immortalize B cells by Epstein-Barr virus using standard protocols [10]. Diagnostic bone marrow samples from acute leukemia patients were received in freezing media from ALL cell bank of Children's Oncology Group (COG) and stored at −80°C. Fetal brain and thymus tissues were collected from 20 week or older fetuses during necropsy and stored at −80°C. cDNAs synthesized from column-purified normal PBMC cell-type subsets, including monocytes (CD14+), NK cells (CD56+, negative selection), B (CD19+) and T lymphocytes (CD3+), were purchased from AllCells, LLC Inc. through StemCell Technologies. Each subset was purified from blood obtained from a different adult subject. The purity and the viability of the each cell-type subset were on average 94% and 97%, respectively, as confirmed by FACScan analyses. The leukemia cell lines were purchased from American Type Culture Collection (ATCC) and grown in the recommended culture conditions to extract DNA and RNA. The B cell lymphoblastic leukemia lines included DB, RL and Pfeiffer. The Pfeiffer cell line contains somatic hypermutations in the rearranged immunoglobulin variable genes [11]. T cell lines included Jurkat, SUP-T1, TALL-104, MOLT-3, MOLT-4, CEM/C2, which were derived from child/adolescent acute leukemia, Loucy, which was derived from adult acute leukemia and HuT 78, HH, which were derived from cutaneous T cell leukemia. NK-92 is an interleukin-2 (IL-2) dependent cell line derived from natural killer cell. U-937, THP-1 and AML-193 were derived from acute monocytic leukemia/lymphoma. All samples were obtained under research protocols reviewed and approved by the University of Pittsburgh Institutional Review Board (IRB) committee.

### Stimulation of PBMCs by phytohemagglutinin (PHA)

PBMCs were freshly isolated from 30 mL peripheral blood drawn into EDTA tubes by Ficoll-Hypaq density-gradient centrifugation. One to two million PBMCs were added to three ml of RPMI 1640 media containing 10% fetal bovine serum, 20 U/ml IL-2 (Pepro Tech) and 2.5 or 5.0 µg/ml of selective T-cell stimulant phytohemagglutinin PHA-L (Roche Diagnostics) and incubated at 37°C and 5% CO_2_ concentration. These recommended PHA concentrations showed robust T cell stimulation, as assessed by MTT cell proliferation kit I (Roche Diagnostics), by inducing activation-induced cell death in the Jurkat cell line. PHA-stimulated PBMCs were sampled at six hours, days 2, 5 and 8 to extract RNA after determining total cell number and fraction of dead cells by trypan blue staining in a hemocytometer. The number of PHA stimulated PBMCs remained unchanged at six hours and day 2 but increased ∼10-fold at days 5 and 8.

### Isolation of CD4+ PBMCs

CD4+ cells were isolated from fresh monocyte-depleted PBMCs, obtained from 30 mL blood, using a positive selection column following the recommended protocol (EasySep™, StemCell Technologies). Monocyte-depleted PBMCs were prepared by Ficoll-Hypaque density separation using RosetteSep™ monocyte (CD36) depletion cocktail (StemCell Technologies).

### RNA isolation, RT-PCR, PCR amplification, plasmid cloning, sequencing

Total RNA was isolated by the single-step method of RNA extraction (RNA-Bee™, Tel-Test). cDNA was synthesized from 0.1–5 µg of total RNA using Maloney Murine Leukemia Virus reverse transcriptase (Life Technologies) and oligo(dT) primers following the recommended protocol. One fifth to one tenth of the freshly synthesized cDNA was used for nested RT-PCR amplification. The steady-state fraction of mutant transcripts was estimated by three semi-independent RT-PCR reactions. For 11 of 179 (6.1%) tested samples, the fraction of mutant transcripts was estimated by two RT-PCR reactions because of assay failure in one of the three replicates. The first round of the nested RT-PCR was performed in three separate reactions using a common forward primer located in exon 1 (F1A) and a unique reverse primer located in exons 3 (R13), 5 (R15) or 8 (R10), respectively (Table S1). The first round PCR products were diluted 1 to 50 in Tris-EDTA (TE) buffer (pH = 8.0) and subjected to second round PCR employing nested primers F1C and R14, which are located in exons 1 and 3, respectively. The first-and second round of RT-PCR employed 30 cycles at 60°C and 61°C annealing temperatures, respectively. By not designing the RT-PCR primers from exon 2, where the R46X mutation is located, co-amplification of the gDNA was prevented. Genomic PCR of *SDHB* exon 2 was performed using intronic primers FN2A, RN2A and 2R. RT-PCR amplification of the other SDH subunit genes, *SDHA*, *SDHC* and *SDHD* were also performed by two rounds of nested PCR using the primers listed in Table S1. To estimate transcript mutation rates (Table S2), RT-PCR products were cloned into plasmid following a commercial protocol (PCR-Script™ Amp, Stratagene) and sequenced by standard automated sequencing after isolating DNAs from plasmids by alkaline miniprep kits (Qiagene). *SDHB* DNA mutation analyses of the leukemic samples were performed by genomic PCR amplification of each of 8 exons and direct sequencing as described [12]. In addition to the novel *SDHB* gene mutations in TALL-104 and Jurkat, we also identified a previously reported silent polymorphism, c.24C>T (Ser8Ser) in the SUP-T1 cell line. The DNA mutations were confirmed by alternative methods including restriction enzyme digestion (for A15T) and cDNA sequencing (for R217H).

Standard PCR amplifications were performed by *Taq* polymerase (GeneChoice). All PCR amplifications were confirmed by gel electrophoresis before further analyses. Each PCR reaction contained 20 pmol of oligonucleotide primers, 1.5 mM of MgCl_2_ in standard 1× PCR buffer, 0.2 mM of each of the dNTPs and 1 Unit of *Taq* polymerase. Each amplification plate had water and TE buffer negative control samples. Both rounds of the RT-PCRs for the assessment of transcript mutation rates (Table S2) and the first round of mutation enrichment RT-PCRs (RT-ePCRs) employed PfuUltra2 enzyme, a high fidelity *Taq* polymerase enzyme, following the recommended protocol (Stratagene).

### Enrichment PCR for the R46X mutation

To enrich for the mutant DNA molecules, we employed enrichment RT-PCR (RT-ePCR) and enrichment genomic PCR (ePCR). RT-ePCR involved overnight-digestion of 10 µL of the first-round PfuUltra-amplified RT-PCR products by 10 U of *Taq*I (New England Biolabs) in a 20 µL reaction volume. The digestion products were then diluted 1 to 10 in TE buffer and PCR amplified in the second round. ePCR started with overnight-digestion of 0.5–1.0 µg of genomic DNA (gDNA) by 10 U of *Taq*I (New England Biolabs) in a 20 µL reaction volume. Four µL of the RE digestion product were then directly used for nested PCR amplifications in two rounds. The first round ePCR products amplified by the intronic primers FN2A and 2R were diluted 1 to 50 in TE buffer and subjected to second round PCR amplification by FN2A and the nested intronic primer RN2A (Table S1).

### Quantification of the R46X mutation by TaqI RE digestion and capillary gel electrophoresis

**Figure 1:**
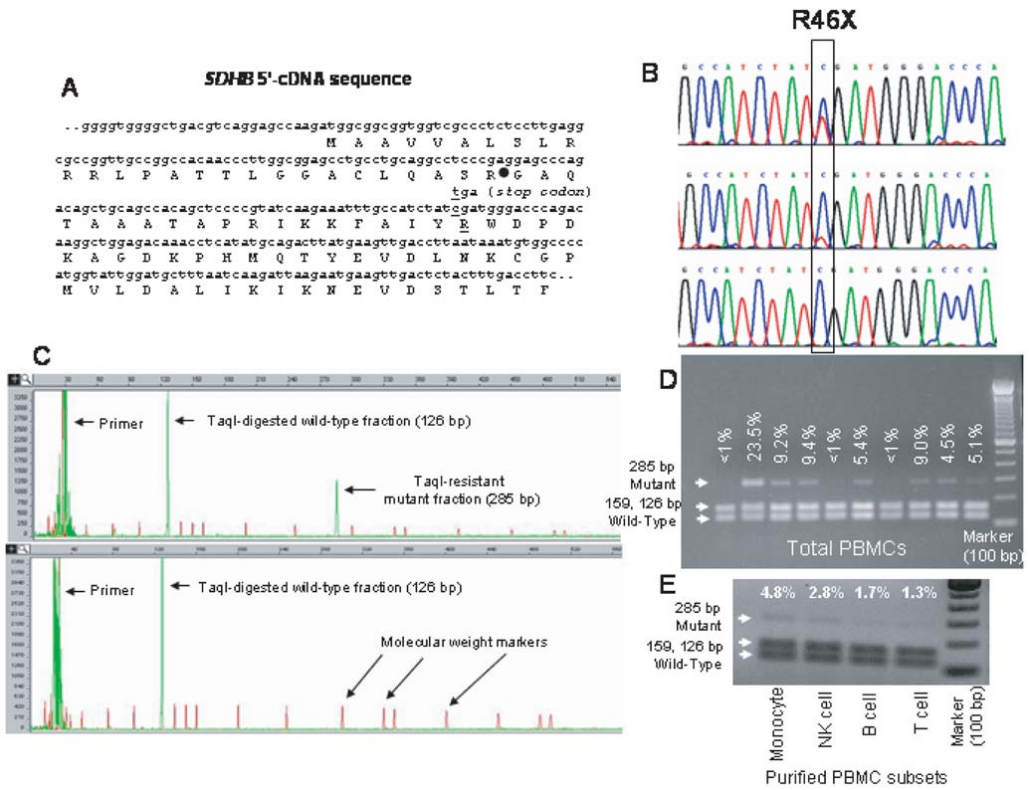
Analysis of *SDHB* R46X mRNA mutation. A. The *SDHB* gene has 843 bp coding nucleotides spread to 8 exons within ∼35 kb at chromosome band 1p36.13. The 5′- portion of the gene shows the position of the R46X mutation and the mitochondrial signal peptide cleavage site (filled circle). B. Sequence chromatograms of RT-PCR products show samples that have high (top), low (middle), or undetectable (bottom) amounts of mutant R46X sequences. C. Quantification of the mutant fraction involved *Taq*I RE digestion of fluorescently-labeled RT-PCR products and capillary gel electrophoresis. The red peaks denote the molecular weight marker. D. The agarose gel electrophoresis shows variable fractions of mutant RT-PCR products from normal PBMCs detected by *Taq*I RE digestion, which releases two bands 159 and 126 bp in size from the wild-type sequence (also see Fig. S1A). E. Fractions of *Taq*I RE resistant transcripts in the purified PBMC cell types are shown in an agarose gel. The negative image is presented to enhance the visibility of mutant transcripts.

The quantification experiments employed the hot-stop PCR method [13]. We removed the PCR plate at the end of the 29^th^ cycle of the second round PCR and added 20 pmol of a HEX-labeled R14 primer (Sigma-Genosys) in a 4 µL 1× PCR buffer. The 30^th^ (last) cycle of the PCR was completed to produce fluorescently-labeled homo-duplex extension products. Fifteen µL of the fluorescently-labeled PCR products, which contained ∼200–300 ng of DNA, were directly digested by 10 U of *Taq*I RE, 1× *Taq*I RE buffer and 1× Bovine Serum Albumin (all from New England Biolabs) overnight in a 25 µL reaction volume. To ensure complete digestion, the reaction was re-dosed with another 10 U of *Taq*I RE in a 5 µL volume and continued for at least another four hours in the following day. All RE digestion products were inspected by gel electrophoresis (Fig. S1). The fluorescently-labeled and *Taq*I-RE digested PCR products were run in capillary gel electrophoresis (3100 Avant Genetic Analyzer, ABI Prism, Applied Biosystems/HITACHI). To quantify the amount of the mutant fraction, the area under the peak for the mutant undigested sequence (285 bp) was divided by the sum of the areas under the peaks for the mutant (undigested) and wild-type (digested, 126 bp) sequences (See Fig. 1C,D).

### Analyses of somatic mutation rates

Nucleotide misincorporation occurs during *in vitro* synthesis of cDNA by reverse transcriptase and PCR amplification. The nucleotide misincorporation rates by Moloney Murine Leukemia virus (MMLV)/SuperScript III reverse transcriptases, *Taq* and PfuUltra DNA polymerases were assumed 6.0×10^−5^, 8×10^−6^ and 0.4×10^−6^ per bp per duplication, respectively [14]. We estimated the highest effective duplication number of *SDHB* cDNAs during the two rounds of nested PCR in PBMCs using control plasmids containing the wild-type gene. Using serial dilutions, we found that after 30 cycles of the first-round PCR, as low as 0.05 fg (0.05×10^−15^ g) of *SDHB* cDNA plasmids did not produce bands detectable in the ethidium bromide-stained agarose gel, similar to the PBMC cDNAs. However, 1 to 50 dilutions of the first-round PCR products yielded ∼400 ng of DNA after the 30 cycle amplification of the second round nested PCR. (*Taq*I RE digestion and gel electrophoreses of the second-round wild-type plasmid PCR products showed no evidence of mutations within the 4-bp RE recognition site introduced during amplification by *Taq* polymerase.) This enrichment corresponds to ∼4×10^11^-fold or ∼38.5 effective duplications by the nested PCR amplification. Consequently, the nested RT-PCR by *Taq* polymerase is expected to generate errors during cDNA synthesis (6.0×10^−5^ per bp) and PCR amplification (38.5×8×10^−6^ per bp), which sums to ∼36.8×10^−5^ per bp (i.e., one error in 2,717 bps). Similarly, nested RT-PCR by PfuUltra is expected to generate a total error rate of ∼7.54×10^−5^ per bp (i.e., one error in 13,263 bp), which was used to test for excess mutations observed in the cloned-sequenced RT-PCR products assuming Poisson distribution (Table S2). Statistical analyses were performed using SISA, a Simple Interactive Package for Statistical Analysis (http://home.clara.net/sisa/index.htm).

### Control experiments on the R46X mutation

#### (a) Overall random mutation rate in SDHB transcripts is not increased in PBMCs

We cloned and sequenced *SDHB* RT-PCR products obtained from total and monocyte-depleted CD4+ PBMCs, leukemic cell lines derived from a post-germinal center B lymphocyte (Pfeifer), a T lymphocyte (Jurkat) and a natural killer cell (NK-92) (Table S2). The R46X mutation was observed among the cDNA clones obtained from total and CD4+ PBMCs and the Jurkat cell line. The overall *SDHB* transcript mutation rate was increased only in the Jurkat line. The mutation rates in the control genes *SDHA*, *SDHD* and *SDHC* were not significantly increased in Jurkat.

#### (b) The R46X mutation is not introduced during enzymatic amplification

Although absence of the R46X mutation in the EBV-transformed lymphoblastoid cell lines strongly suggests that the mutation is not introduced during *in vitro* amplification, to further rule out this possibility, we also tested wild-type *SDHB* plasmids. On the basis of nucleotide misincorporation rates, enzymatic amplification of the wild-type plasmids for 38.5 effective duplications during nested PCR (above) are expected to generate random mutations in ∼0.15% of the PCR products within the 4 bp-long *Taq*I RE recognition site. Accordingly, agarose and capillary gel electrophoreses showed no evidence of mutations in the *Taq*I RE recognition site in the PCR products amplified from 10^6^-fold range of starting wild-type plasmid DNA amounts (Fig. S1). When wild-type *SDHB* plasmids were PCR amplified in the first-round, digested by *Taq*I RE and subjected to a second-round PCR (i.e., mutation enrichment PCR, ePCR), no plasmid template (n = 19) showed the R46X mutation by direct sequencing. In contrast, all PBMC cDNAs (n = 35) tested by RT-ePCR showed the R46X mutation by direct sequencing (Fig. S1B). Finally, the R46X mutation was observed after RT-PCR amplification either by *Taq* polymerase or by PfuUltra (Table S2) and after reverse transcription either by MMLV or by SuperScript III (Life Technologies) reverse transcriptase enzymes.

#### (c) Nested PCR amplification does not significantly distort the starting fractions of mutant cDNAs

To test whether nested PCR amplification of *SDHB* significantly distorts the starting fraction of mutant cDNAs, we amplified control plasmid templates which contained pre-determined fractions of mutant DNAs and quantified the percentage of mutant molecules by *Taq*I RE digestion and capillary gel electrophoresis. Primers F1C-R10, F1C-R15 and F1C-R13 were used in the first-round of the nested PCR to generate three replicates for each sample. Each replicate was diluted 1 to 50 in T.E. buffer and then PCR amplified by F1C-R14 in the second-round, in the last cycle of which a fluorescently-labeled R14 primer was added. The confidence intervals of the expected and the observed percentages of mutant DNAs overlapped over a 1,000-fold range of starting fractions of the mutant DNA (Fig. S2[Supplementary-material pone.0000436.s002]) suggesting that nested PCR does not significantly skew the fraction of mutant DNAs.

#### (d) The R46X mutation targets the full-length SDHB coding mRNA sequences

Northern analysis indicated that *SDHB* is ubiquitously expressed and its transcripts cluster at a single band at ∼1.1 kb size (Fig. S3). RT-PCR, *Taq*I RE digestion and gel electrophoresis of PBMC mRNAs showed that R46X occurs in the full-length *SDHB* coding mRNA. This is also confirmed by direct sequencing of 23 clones that have the R46X mutation.

#### (e) No processed SDHB pseudogene exists in the gDNA

Processed pseudogenes in the gDNA could co-amplify during RT-PCR and potentially complicate interpretation of cDNA-based mutation analyses. Sequence of the human genome does not indicate presence of a pseudogene for *SDHB* (http://www.genome.ucsc.edu). Accordingly, PCR amplification of gDNA by *SDHB* RT-PCR primers F1A and R13 did not yield any products in 23 samples (data not shown).

## Results

### Discovery of SDHB R46X mRNA mutation

While investigating consequences of a PGL-associated *SDHB* splice-site mutation IVS1 +1 G>T on transcript splicing in an affected subject by reverse transcription, nested PCR amplification (RT-PCR) and direct sequencing we found that a fraction of the correctly-spliced *SDHB* complementary DNAs (cDNAs) synthesized from peripheral blood mononuclear cells (PBMCs) contained a premature termination codon mutation, c.136C>T (R46X) (Fig. 1A) that was absent in the genomic DNA (gDNA). The R46X transcript mutation was subsequently identified in several unaffected subjects in variable fractions by direct sequencing of PBMC cDNAs (Fig. 1B). The R46X, a pathogenic mutation in exon 2, has been previously identified in the germ lines of multiple PGL cases [3] and is predicted to truncate the *SDHB* protein product shortly after the mitochondrial signal peptide sequence (Fig. 1A). Further control experiments, which are explained in detail in Methods, showed that (a) overall random mutation rate in *SDHB* transcripts is not increased in PBMCs (b) the R46X mutation is not introduced by *Taq* polymerase; (c) Nested PCR amplification does not significantly distort the starting fractions of mutant cDNAs; (d) the R46X mutation targets the full-length *SDHB* coding mRNA sequences; (e) no *SDHB* pseudogene exists in the gDNA.

### Quantification of the R46X mutation in PBMCs

**Figure 2:**
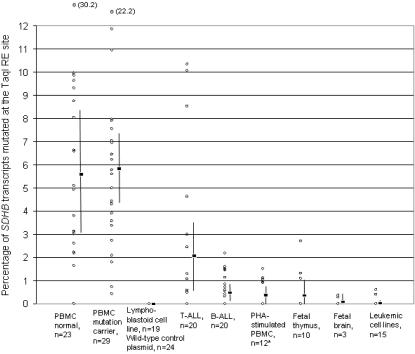
The steady-state fraction of mutant *SDHB* cDNAs resistant to *Taq*I RE digestion. Each circle represents a sample from a different subject (total indicated by n) except for those in the PHA stimulated PBMCs which were obtained from two normal donors, stimulated by 2.5 µg/ml and 5.0 µg/ml concentrations of PHA and tested at days 2, 5 and 8 for a total of 12 samples. The value corresponding to each circle was derived from three RT-PCR reactions. Two outlier PBMC values were shown at the top with their mutant transcript fractions in parentheses. Boxes and the vertical lines denote the means and their 95% confidence intervals of samples sets. “PBMC mutation carrier” group contains 5 *SDHC* and 24 *SDHD* mutation carriers. The leukemic cell lines were derived from B cells (n = 3), T cells (n = 9), NK cells (n = 1) and monocytes (n = 2).

To quantify the fraction of *SDHB* transcripts containing R46X, we screened a unique *Taq*I restriction enzyme (RE) recognition site (TCGA) destroyed by the mutation using fluorescent “hot-stop” RT-PCR, *Taq*I RE digestion and capillary gel electrophoresis (Fig. 1C, D). The average fraction of the R46X-containing *SDHB* transcripts obtained from PBMCs of normal controls and of *SDHD/C* germ line mutation carriers were 5.7% and 5.9% (range less than 1 to 30%), respectively (Fig. 2). Cloning-sequencing confirmed that ∼99% of the *Taq*I-RE-digestion-resistant RT-PCR clones from PBMCs were composed of the R46X mutation (Table 1). When the second round RT-PCR was performed after removing the wild-type sequences from the first round products by *Taq*I RE digestion (i.e., enrichment RT-PCR; RT-ePCR), all tested PBMCs showed the R46X mutation by direct sequencing (see Methods). Thus, the average inactivation rate of *SDHB* mRNAs in PBMCs is ∼10,000-fold higher than would be expected from all background *in vivo* somatic inactivating DNA mutations as measured in the *HGPRT* gene in normal adult peripheral blood T lymphocytes [15]. The levels of the mutated *SDHB* transcripts in PBMCs are comparable to the C-to-U-edited neurofibromatosis 1 (*NF1*) mRNAs that occur in substantial levels (>3%) in certain NF1-related tumors, but not in normal tissues, and are thought to be catalyzed by the apolipoprotein B mRNA-editing enzyme APOBEC-1 [16].

**Table 1:** Sequence profiles of *Taq*I-RE-digestion-resistant mutant *SDHB* clones

Nucleic acid source (n = number of samples)	Nucleic acid type/PCR method	No. of clones TTGA *R46X*	No. of clones TCGG	No. of clones TCAA	No. of clones CCGA	No. of clones ACGA	No. of clones TAGA	No. of clones TCGT	Single clones	Total no. of clones	P for enrichment of TTGA clones&
PBMC (n = 6)	cDNA/RT-PCR	92	0	0	0	0	0	0	TTGG	93	∼0
Pure Monocytes (n = 1)	cDNA/RT-PCR	32	2	0	2	0	0	0	TGGA	37	∼0
T-ALL primary (n = 3)	cDNA/RT-PCR	75	13	4	5	0	0	0	TGGA	98	∼0
T-ALL cell lines*	cDNA/RT-ePCR	27	7	6	9	3	0	1	ATAG	54	2.2×10^−4^
NK-92 (NK cell)	cDNA/RT-ePCR	2	1	2	10	8	0	3	0	26	0.33
Pfeiffer (B cell)	cDNA/RT-ePCR	2	2	0	16	0	0	3	0	23	0.33
THP-1 (Monocyte)	cDNA/RT-ePCR	9	10	13	14	4	3	1	TCACTCTA	56	0.60
PBMC (n = 3)	gDNA/ePCR	27	0	0	0	0	2	0	TGGA	30	∼0
T-ALL cell linesˆ	gDNA/ePCR	43	0	1	3	0	6	0	TCTA	54	∼0

* Includes Jurkat, Loucy, SUP-T1, HuT 78.

ˆ Includes MOLT-4 and Jurkat.

& Fisher's exact test of the hypotheses that number of TTGA clones should be ∼16% of the total on the basis of random somatic mutation data [15].

### Analyses of the R46X mutation in PBMC cell-type subsets

Because PBMCs are primarily composed of lymphocytes and monocytes, we analyzed cDNAs obtained from purified peripheral blood monocytes, natural killer (NK) cells, T and B lymphocytes. The fraction of mutant transcripts was higher in monocytes and NK cells than in T and B lymphocytes (Figure 1E). Cloning-sequencing of *Taq*I-RE-digestion-resistant band from the monocytes confirmed the R46X mutation in 32 of 37 (86%) clones (Table 1). Mutation enrichment RT-PCR (RT-ePCR) analyses confirmed the presence of R46X mutation in each of the PBMC cell-type subset (data not shown).

### Analyses of the R46X mutation in leukemic cell lines

To test presence of the R46X transcript mutation in leukemic cell lines derived from PBMCs, we amplified *SDHB* cDNAs mutated at the *Taq*I RE site by RT-ePCR. Cloning-sequencing of *Taq*I-RE-digestion-resistant products showed the R46X mutation in 27 of 54 (50%) clones in the T cell leukemia cell lines but only in 2 of 26 (7.7%) clones in NK-92, which is derived from an NK cell, in 2 of 23 (8.7%) in Pfeiffer, which is derived from a post-germinal center B lymphocyte, and in 9 of 56 (16.1%) of THP-1, which is derived from a monocyte (Table 1). The number of clones with the R46X mutation was statistically significantly overrepresented only in the T-ALL cell lines relative to the random somatic mutation profiles [15] (Table 1). Furthermore, the overall transcription mutation rate in *SDHB* but not *SDHA*, *SDHC* and *SDHD* was increased in the Jurkat T cell line (Table S2).

### Analyses of the R46X mutation in primary leukemic and various other samples

Next, we compared the *Taq*I-resistant mutant transcript fractions in a total of 175 samples including EBV-transformed lymphoblastoid cell lines, which are derived from peripheral blood B cells, various commercial leukemic cell lines (see Methods), diagnostic bone marrow samples from childhood T- and B-ALL, PBMCs treated by T cell-stimulant phytohemagglutinin (PHA), fetal thymus and brain tissues and non-mutant wild-type plasmid control DNAs. Substantial levels (>3%) of *Taq*I-RE-digestion-resistant mutant transcripts were observed in five of 20 diagnostic primary T-ALL samples (Fig. 2). Cloning-sequencing of the *Taq*I-digestion resistant bands from T-ALL primary samples uncovered the R46X mutation in 27 of 43 (62%) clones (Table 1). Because primary diagnostic leukemic bone marrow samples are significantly enriched for the lineage-specific lymphocytes, these results further suggest that the mutational mechanism targeting the *SDHB* mRNA remains functional in leukemic T cells.

### Analyses of the R46X mutation in genomic DNA (gDNA)

**Figure 3:**
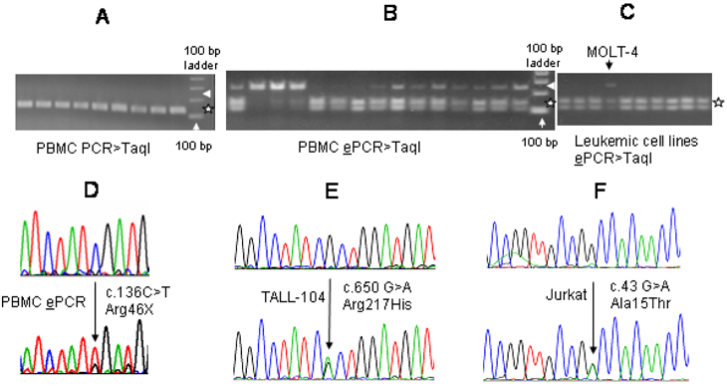
*SDHB* mutations in genomic DNAs (gDNAs). A. *Taq*I RE digestion of PCR-amplified exon 2 shows no evidence of undigested mutant DNAs at 260 bp size (pointed by an arrow head). *Taq*I RE digestion products of the wild-type DNA co-migrate at sizes of 128 and 132 bps (denoted by a star). B. Gel electrophoresis of *Taq*I RE-digested (mutation) enrichment PCR (ePCR) products shows variable amounts of mutant gDNAs at 233 bp size (pointed by an arrow head), which are confirmed to be primarily composed of c.136C>T by sequencing (Table 1). *Taq*I digestion of wild-type ePCR products gives two bands at sizes, 101 and 132 bp (shown by a star). C. ePCR analyses of leukemic cell lines demonstrate the presence of mutant gDNA in the MOLT-4 cell line (pointed by an arrow head) but not in other leukemic cell lines in this experiment. D. Direct sequence analysis of a gDNA ePCR product shows selective enrichment of the R46X mutation. E, F. Sequence chromatograms of the heterozygous DNA mutations detected in two T-ALL cell lines are shown.

To test whether the R46X mutation also occurs in the gDNA of PBMCs, we initially PCR-amplified *SDHB* exon 2 from 22 samples and digested it with *Taq*I RE. Gel electrophoresis of the RE digestion products showed no evidence of the mutation (Fig. 3A), including in the sample that showed the highest fraction of the mutant cDNA (30.2% in Fig. 2). To test whether the R46X mutation exists in the gDNA at very low levels, we employed enrichment PCR (ePCR), which involved removal of the non-mutant wild-type gDNA by *Taq*I RE digestion before nested PCR amplification. Analyses of the ePCR products by *Taq*I RE digestion and gel electrophoresis showed variable fractions of mutant molecules in PBMC gDNAs (Fig. 3B). Direct sequencing of ePCR products showed the R46X mutation in 3 of 24 samples (Fig. 3D). Cloning-sequencing of the *Taq*I RE digestion-resistant ePCR products from these three samples showed the R46X mutation in 27 of 30 clones (90%) confirming that the ePCR method statistically significantly enriched for the R46X mutation (Table 1). These findings indicate that the R46X mutation identified in *SDHB* cDNA also occurs in the gDNA at a much lower frequency.

To test whether genomic R46X mutation also occurs in the leukemic cell lines, we employed ePCR on the gDNAs of 11 leukemic cell lines including four originating from T cells (Jurkat, CEM/C2, Loucy, MOLT-4), three from B cells (Pfeiffer, DB and RL), three from monocytes (AML-93, U-937, THP-1) and one from NK cells (NK-92). Analyses of the ePCR products by *Taq*I RE digestion and gel electrophoresis uncovered the presence of mutant bands in the CD4+ T-ALL cell lines MOLT-4 and Jurkat in three replicate experiments (Fig. 3C). Cloning-sequencing of the *Taq*I RE digestion-resistant bands from MOLT-4 and Jurkat uncovered R46X in 43 of 54 clones (∼80%) (Table 1), demonstrating a statistically significant enrichment of the mutation. These results further suggest that the R46X mutation occurs in leukemic T lymphocytes.

### SDHB gene sequencing in PBMC-derived leukemic cell lines

We sequenced all eight *SDHB* gene exons in 16 leukemic cell line DNAs, including those derived from T, B, NK cells and monocytes, and found novel expressed DNA sequence alterations in two of six childhood T-ALL cell lines. The Arg217His mutation in TALL-104 (Fig. 3E) targets an amino acid residue conserved in 497 of 500 *SDHB* sequence entries from diverse organisms in GenBank and its distinct germ line missense mutation, Arg217Cys, is previously linked to PGL susceptibility [3]. The Ala15Thr mutation in Jurkat (Fig. 3F) decreases hydrophocity of the mitochondrial signal peptide by converting a non-polar to a polar residue. The mutated alanine is conserved in 20 of 23 multicellular eukaryotic organisms which have the orthologous signal peptide sequences including fly, chicken and mammals (Fig. S4). These two missense variants were not present in the *SDHB* orthologous amino acid positions in other organisms in GenBank, in over 200 human chromosomes that we previously sequenced, and have not been reported as population variants in extensive mutation surveys of PGL [3]. No *SDHB* gDNA mutations were detected in 20 T-ALL primary diagnostic samples.

## Discussion

The current study describes identification of substantial levels of *SDHB* transcripts carrying a recurrent R46X mutation in normal PBMCs. Analyses of the purified PBMC cell-types suggest monocytes and NK cells as the major sources of the mutant transcripts, although T and B lymphocytes also contribute at lower levels. In contrast, analyses of leukemic cell lines and bone marrow samples uncover the R46X transcripts only in T-ALL. The R46X-mRNAs constitute on average 5.8% of all *SDHB* transcripts in PBMCs, strongly suggesting that a cytidine deaminase enzyme(s) catalyzes this somatic mutation.

Programmed somatic mutations targeting cytidine residues in protein-encoding mammalian nuclear genes were previously observed in two normal cell types: the post-germinal center B lymphocytes during diversification of immunoglobulins by the process of somatic hypermutation [17] and in intestinal cells to generate a shorter protein isoform (ApoB48) of apolipoprotein B by RNA editing [18]. The mutations in the immunoglobulin and apolipoprotein B loci confer new properties to the targeted gene products (i.e., gain-of-function). The R46X mutation in *SDHB* is unusual because it predicts loss-of-function, it targets a classical tumor-suppressor gene, and it occurs in PBMCs.

The results described here prompt important questions on mechanism and significance of *SDHB* mRNA mutations identified in normal PBMCs and leukemic T cells. The current results show that the R46X mutation is present at much higher proportions in mRNA than in DNA. This finding could be explained by two models. First, the putative mutator enzyme may target both DNA and mRNA, the latter with a higher efficiency. Alternatively, the mutator enzyme may target only DNA, which is then repaired after the transcription. The presence of sequence heterogeneity and A/T mutations in the *Taq*I RE recognition site (e.g., in the T-ALL bone marrow samples in Table 1) supports the second model because such sequence variants suggest operation of an error-prone repair mechanism in the leukemic T cells. The error-prone DNA repair of uracil residues is thought to be responsible for the generation of A/T mutations during antibody diversification in B cells by the activation-induced cytosine deaminase (AID) enzyme [19].

Another important question concerns the causes of variation in the fraction of R46X-mRNAs obtained from total PBMCs. Heritable or acquired conditions such as infections, medications, exercise and diurnal rhythm might influence the relative abundance of PBMC cell-type subsets and, consequently, the fraction of the R46X-mRNAs. Alternatively, technical factors such as the length of time span and the storage conditions between the blood draw to the PBMC isolation procedure might affect the cell viability. Furthermore, if the R46X-mRNAs are variably targeted by nonsense-mediated decay, an intra-cellular surveillance mechanism that removes the transcripts containing premature termination codons [20], the steady-state fraction of mutant *SDHB* mRNAs would underestimate the true fraction of mutated transcripts in certain samples or PBMC cell-type subsets. Although each of these factors might influence the proportion of mutated *SDHB* mRNAs in a given sample, the present results indicate that the R46X mRNA mutation occurs in every tested PBMC sample at significantly higher levels compared to the control lymphoblastoid cell lines or wild-type plasmid templates. The identification of distinct heterozygous missense *SDHB* gene mutations in two T-ALL cell lines further suggests that the R46X mutation might play a functional role in the biology of PBMCs and leukemic T cells.

**Figure 4:**
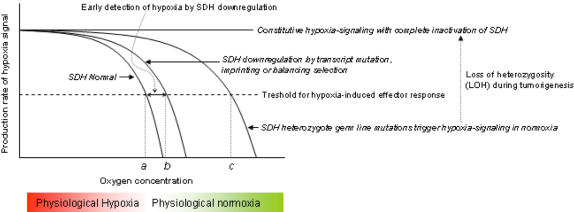
SDH genetic downregulation as a mechanism for early detection of hypoxia. Data from PGL research suggest that SDH normally inactivates a messenger(s) critical for downstream hypoxia-signaling. Consequently, heterozygous SDH germ line mutations over-activate the hypoxia signaling pathways in PGL. Loss of the wild-type allele by loss-of-heterozygosity (LOH) leads to constitutive hypoxic signaling and tumorigenesis in PGL. Transcript mutations, genomic imprinting, balancing selection or germ line mutations are postulated to reduce SDH activity and change the hypoxia-detection threshold from a lower oxygen concentration (*a*) to higher oxygen concentrations (*b, c*), thus leading to earlier detection of impending hypoxia.

The genetic regulation of SDH subunits appears to be emerging as a common motif in several normal cell types. First, the transmission pattern of PGL1 tumors suggests that the *SDHD* subunit gene is suppressed on the maternal allele by genomic imprinting in the paraganglionic tissues [2]. The *SDHD* mutations or chronic hypoxic exposure cause paragangliomas in the carotid body (CB). Notably, the CB is the major oxygen sensor organ in mammals and stimulates the cardiopulmonary system within seconds of exposure to hypoxia. Second, the *SDHA* gene, which encodes the major catalytical subunit (flavoprotein) of SDH, carries a strong signature of balancing selection in the African chromosomes [21]. Distinct *SDHA* alleles, including two missense variants at amino acid residues conserved in mammals, have been maintained at intermediate frequencies for long evolutionary periods. Thus, certain *SDHA* allelic variants that have slight functional deficits might confer protection against pathogens or toxins that are prevalent in Africa. Finally, the present results demonstrate a recurrent *SDHB* mutation in normal PBMCs and leukemic T cells. Although many gaps exist in our knowledge on the mechanisms of imprinting on *SDHD*, balancing selection on *SDHA* and transcript mutations in *SDHB*, including their impact on SDH enzyme activity, the extant data on PGL suggest that these regulatory mechanisms might downregulate SDH function and facilitate early detection of and pre-adaptation to hypoxia (Figure 4).

It is well-known that blood monocytes differentiate into macrophages in solid tissues and accumulate in large numbers in chronically hypoxic avascular and necrotic areas in infected and neoplastic organs [22], [23]. The *SDHB* somatic transcript mutations might enable earlier activation of hypoxia inducible pathways and increase survival of monocytes and natural killer cells in these hypoxic areas. A similar survival advantage might be conferred to leukemic T cells as they shuttle between the well-oxygenated peripheral blood and the more hypoxic immune-system organs. In conclusion, the present results suggest that the *SDHB* gene is targeted by a somatic mutational mechanism that likely involves the activity of a cytidine deaminase enzyme expressed in PBMCs and leukemic T cells. If the somatic *SDHB* mutations are proven to play a role in oxygen homeostasis in PBMCs and leukemic T cells, targeting of the SDH complex might provide new therapeutic tools against infections, leukemia and cancer.

## Supporting Information

Figure S1 Detecting the R46X mutation in agarose gel electrophoresis. Each test set contains 16 different samples. The second-round RT-PCR products remaining undigested after the TaqI RE digestion at 285 bp (examples shown by arrows) indicate the presence of mutations within the 4-bp RE recognition. Highest levels of mutations were observed in the PBMC and T-ALL samples (also see Figure 2). Plasmid controls demonstrate outcome of the experiment when plasmids (∼4.0 kb) containing the wild-type SDHB cDNA were used for nested PCR amplification at various starting amounts shown by numbers 1 to 4. Total plasmid input amounts: 1 = 28 ng, 2 = 0.28 ng, 3 = 2.8 pg, 4 = 28 fg. B. Enrichment of R46X by enrichment RT-PCR (RT-ePCR) in PBMCs. RT-ePCR involved removal of the wild-type sequences by TaqI digestion before the second-round PCR. Direct sequencing of second-round RT-ePCR products confirmed presence of the R46X mutation in all tested PBMCs (n = 35) but in none of the control wild-type plasmid templates (n = 19) nor in the lymhoblastoid cell lines (n = 14).
(0.18 MB TIF)

Figure S2 Fraction of mutant cDNAs before and after PCR amplification. Mutant and wild-type plasmid DNAs that have full-length SDHB cDNAs were mixed in variable amounts to generate control template sets (denoted by letters A–I) for nested PCR. (Image for set E was re-positioned at the end of other sets from the lower half of the gel.) Each template set was composed of two samples (shown by numbers 1 and 2) that have the same fraction of mutant DNAs but different starting amounts of total plasmid [∼20 and 5 fg (10–15 g), respectively]. The top of the figure shows Taq I RE digestion results of the second round PCR products that were amplified by F1C-R10, F1C-R15 (shown under the gel pictures) in the first-round and F1C-R14 in the second round. Graph at the bottom shows, on a logarithmic scale, the average percentages (denoted in parentheses) of the expected (starting) and observed (measured after nested PCR) ratios of mutant/wild type plasmids in the test sets. The confidence intervals (delimited by plus signs) for the expected percentages were derived from the most extreme values of plasmid DNA concentrations that were obtained from multiple (n = 7) spectrophotometric measurements. For the observed percentages, 95% confidence intervals were derived from quantification of the six replicates in each set. The lower boundaries of the observed confidence intervals for sets G, H were zero. Set I, which is not shown in the graph, has 0.02% expected and 0% observed mutant DNAs, respectively.
(0.09 MB TIF)

Figure S3 Northern analysis of SDHB mRNA. SDHB is ubiquitously expressed and its transcripts cluster at a single band of ∼1.1 kb size. Multiple Tissue Northern (MTN™, CLONTECH) Blot contained ∼2 microgram of mRNA in each lane. The hybridization probe was generated by RT-PCR amplification of the full-length SDHB gene by primers F1A-R9 (Table S1) and labeled by 32P following the random priming method using a commercial protocol (High Prime, Roche).
(0.05 MB TIF)

Figure S4 The Ala15Thr mutation in Jurkat cell line. Multiple sequence alignment (Clustal W 1.83) of N-terminal sequences of SDHB gene products demonstrates that human Ala15 is conserved (shown by red fonts) in most organisms which are denoted by their genus names. All sequences detected by the default parameters of BLAST analyses (http://www.ncbi.nlm.nih.gov/BLAST) are shown.
(0.05 MB TIF)

Table S1 Oligonucleotide PCR primers used to amplify SDH subunit genes by nested PCR. * Primers used in both rounds of the nested PCR.
(0.04 MB DOC)

Table S2 Transcript mutations in SDH subunit genes. *1 misincorporation per 13,263 bp on the basis of RT-PCR amplification by PfuUltra. Ns = not significant
(0.04 MB DOC)
