# Supplementary figures and images for: A Recurrent Stop-Codon Mutation in Succinate Dehydrogenase Subunit B Gene in Normal Peripheral Blood and Childhood T-Cell Acute Leukemia
Source: PLoS One. 2007 May 9;2(5):e436. doi: 10.1371/journal.pone.0000436 (PMC1855983; doi:10.1371/journal.pone.0000436)

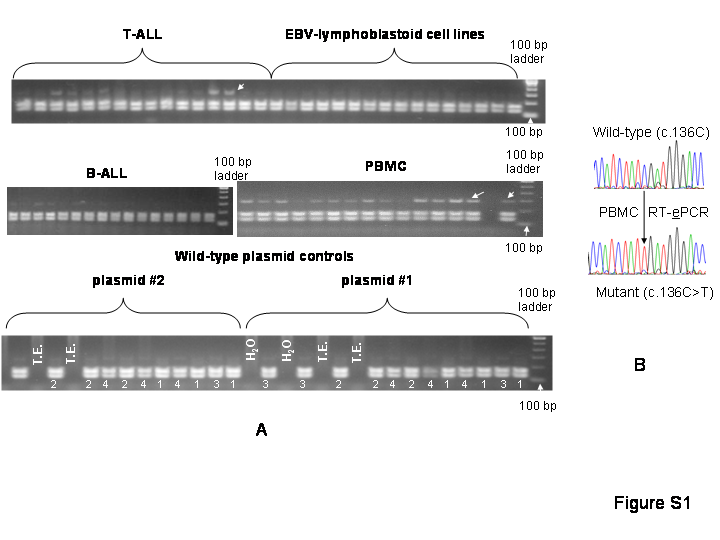

Supplement: Figure S1 — Detecting the R46X mutation in agarose gel electrophoresis. Each test set contains 16 different samples. The second-round RT-PCR products remaining undigested after the TaqI RE digestion at 285 bp (examples shown by arrows) indicate the presence of mutations within the 4-bp RE recognition. Highest levels of mutations were observed in the PBMC and T-ALL samples (also see Figure 2). Plasmid controls demonstrate outcome of the experiment when plasmids (∼4.0 kb) containing the wild-type SDHB cDNA were used for nested PCR amplification at various starting amounts shown by numbers 1 to 4. Total plasmid input amounts: 1 = 28 ng, 2 = 0.28 ng, 3 = 2.8 pg, 4 = 28 fg. B. Enrichment of R46X by enrichment RT-PCR (RT-ePCR) in PBMCs. RT-ePCR involved removal of the wild-type sequences by TaqI digestion before the second-round PCR. Direct sequencing of second-round RT-ePCR products confirmed presence of the R46X mutation in all tested PBMCs (n = 35) but in none of the control wild-type plasmid templates (n = 19) nor in the lymhoblastoid cell lines (n = 14). (0.18 MB TIF) [file pone.0000436.s001.tif]

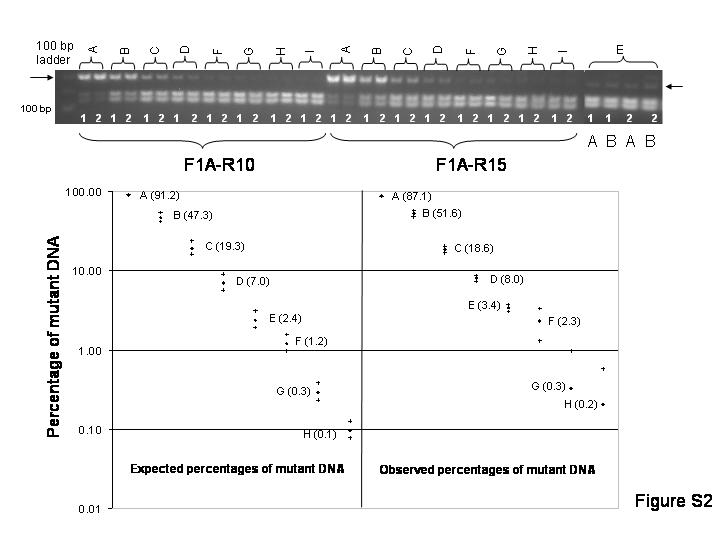

Supplement: Figure S2 — Fraction of mutant cDNAs before and after PCR amplification. Mutant and wild-type plasmid DNAs that have full-length SDHB cDNAs were mixed in variable amounts to generate control template sets (denoted by letters A–I) for nested PCR. (Image for set E was re-positioned at the end of other sets from the lower half of the gel.) Each template set was composed of two samples (shown by numbers 1 and 2) that have the same fraction of mutant DNAs but different starting amounts of total plasmid [∼20 and 5 fg (10–15 g), respectively]. The top of the figure shows Taq I RE digestion results of the second round PCR products that were amplified by F1C-R10, F1C-R15 (shown under the gel pictures) in the first-round and F1C-R14 in the second round. Graph at the bottom shows, on a logarithmic scale, the average percentages (denoted in parentheses) of the expected (starting) and observed (measured after nested PCR) ratios of mutant/wild type plasmids in the test sets. The confidence intervals (delimited by plus signs) for the expected percentages were derived from the most extreme values of plasmid DNA concentrations that were obtained from multiple (n = 7) spectrophotometric measurements. For the observed percentages, 95% confidence intervals were derived from quantification of the six replicates in each set. The lower boundaries of the observed confidence intervals for sets G, H were zero. Set I, which is not shown in the graph, has 0.02% expected and 0% observed mutant DNAs, respectively. (0.09 MB TIF) [file pone.0000436.s002.tif]

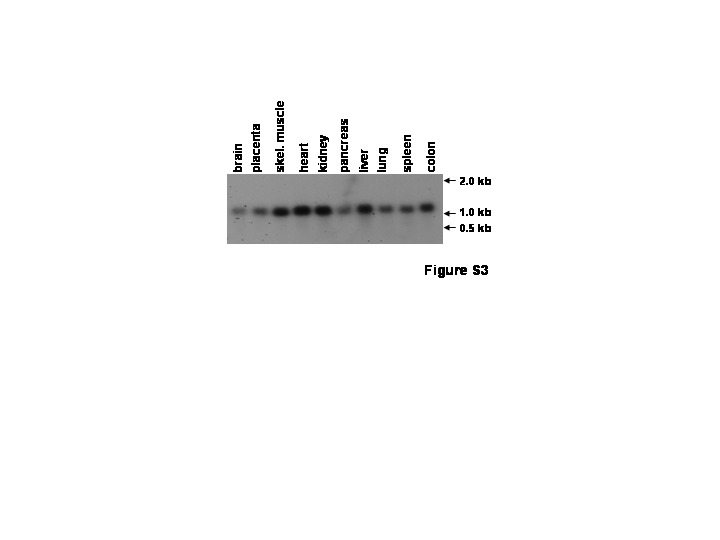

Supplement: Figure S3 — Northern analysis of SDHB mRNA. SDHB is ubiquitously expressed and its transcripts cluster at a single band of ∼1.1 kb size. Multiple Tissue Northern (MTN™, CLONTECH) Blot contained ∼2 microgram of mRNA in each lane. The hybridization probe was generated by RT-PCR amplification of the full-length SDHB gene by primers F1A-R9 (Table S1) and labeled by 32P following the random priming method using a commercial protocol (High Prime, Roche). (0.05 MB TIF) [file pone.0000436.s003.tif]

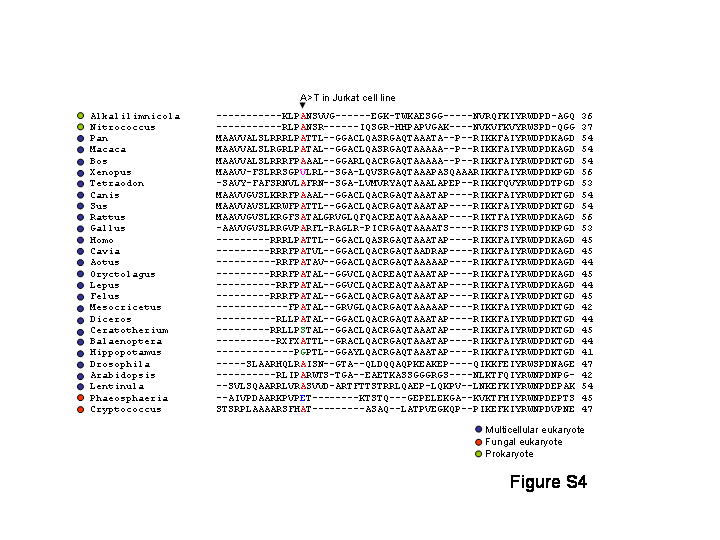

Supplement: Figure S4 — The Ala15Thr mutation in Jurkat cell line. Multiple sequence alignment (Clustal W 1.83) of N-terminal sequences of SDHB gene products demonstrates that human Ala15 is conserved (shown by red fonts) in most organisms which are denoted by their genus names. All sequences detected by the default parameters of BLAST analyses (http://www.ncbi.nlm.nih.gov/BLAST) are shown. (0.05 MB TIF) [file pone.0000436.s004.tif]
