# Supplementary material for: A Recurrent Stop-Codon Mutation in Succinate Dehydrogenase Subunit B Gene in Normal Peripheral Blood and Childhood T-Cell Acute Leukemia
Source: PLoS One. 2007 May 9;2(5):e436. doi: 10.1371/journal.pone.0000436 (PMC1855983; doi:10.1371/journal.pone.0000436)
Supplement: Table S1 — Oligonucleotide PCR primers used to amplify SDH subunit genes by nested PCR. * Primers used in both rounds of the nested PCR. (0.04 MB DOC) [file pone.0000436.s005.doc]

**Table S1**

**Oligonucleotide PCR primers used to amplify SDH subunit genes by nested PCR**

| **Gene** | **Primer name (PCR round used)** | **primer location** | **Sequence (5’ to 3’)** |
| --- | --- | --- | --- |
| *SDHB* | F1A (1) | exon 1 | GGTCCTCAGTGGATGTAGGC |
|  | F1C (2) | exon 1 | GGTGGGGCTGGACGTCAGGA |
|  | R9 (1) | exon 8 | ATGTTCAGCTCTGAGCTGGTT |
|  | R10 (1,2)* | exon 8 | ACCAAGATCTTTAAAGGAACTCA |
|  | R15 (1) | exon 5 | TGGACTGCAGATACTGCTGCT |
|  | R13 (1) | exon 3 | TCTGCATGATCTTCGGAAGGT |
|  | R14 (2) | exon 3 | CAAAGTAGAGTCAACTTCATTC |
|  | FN2A (1,2)* | intron 1 | CCAGCAAAATGGAATTATCTTGT |
|  | R2 (1) | intron 2 | AAGCATGTCCCTAAATCAAA |
|  | RN2A (2) | intron 2 | CTCTCCTTCAATAGCTGGCTT |
| *SDHD* | F1D (1) | exon 1 | TCGTCGTCGTGGGTGGGAA |
|  | R3A (1) | exon 3 | GACCATGAAGAGTGAGGGC |
|  | F1E (2) | exon 1 | ATTGTCGCCTAAGTGGTTCC |
|  | R3B (2) | exon 3 | CAGCCGGAAGCAGACCCA |
| *SDHA* | F1 (1) | exon 3 | ACTGCGCGGCGGCAACAGCA |
|  | R15A (1) | exon 15 | CTGGCAAGCTCCCAGCCA |
|  | F2 (2) | Exon 5 | TTGGTGGACAGAGCCTCAAGTT |
|  | R3 (2) | Exon 10 | TGCTGAGTCGCAGTTCCGAT |
| *SDHC* | F1 (1,2)* | exon 1 | CACTTCCGTCCAGACCGGA |
|  | R6A (1) | exon 6 | TACTCTACTGCTCCAAGGA |
|  | R6B (2) | exon 6 | ATGATGCTGGGAGCCTCCT |

* Primers used in both rounds of the nested PCR.
