# Supplementary material for: A Recurrent Stop-Codon Mutation in Succinate Dehydrogenase Subunit B Gene in Normal Peripheral Blood and Childhood T-Cell Acute Leukemia
Source: PLoS One. 2007 May 9;2(5):e436. doi: 10.1371/journal.pone.0000436 (PMC1855983; doi:10.1371/journal.pone.0000436)
Supplement: Table S2 — Transcript mutations in SDH subunit genes. *1 misincorporation per 13,263 bp on the basis of RT-PCR amplification by PfuUltra. Ns = not significant (0.04 MB DOC) [file pone.0000436.s006.doc]

**Table S2**

**Transcript mutation**s in SDH subunit genes

| mRNA source | Gene | Base pairs  analyzed | Expected no. of mutations^*^ | Observed  no. of mutations | *P* (Poisson) for overall mutation rate | No. of *SDHB* clones with R46X/all clones containing the site | No. of clones with mutations/all clones |
| --- | --- | --- | --- | --- | --- | --- | --- |
| Normal PBMCs  (n=3) | *SDHB* | 19,389 | 1.5 | 2 | Ns | 2/23 | 2/23 |
| Blood CD4+ cells  (n=3) | *SDHB* | 31,421 | 2.4 | 3 | Ns | 1/27 | 3/44 |
| Jurkat (T cell) | *SDHB* | 17,745 | 1.3 | 7 | 4.8 x 10^-4^ | 1/37 | 3/38 |
| Pfeiffer (B cell) | *SDHB* | 3,936 | 0.3 | 0 | Ns | 0/16 | 0/16 |
| NK-92 (NK cell) | *SDHB* | 30,800 | 2.3 | 2 | Ns | 0/25 | 2/41 |
| Jurkat (T cell) | *SDHA* | 16,348 | 1.2 | 0 | Ns | - | 0/20 |
| Jurkat (T cell) | *SDHD* | 5,235 | 0.4 | 0 | Ns | - | 0/22 |
| Jurkat (T cell) | *SDHC* | 9,003 | 0.7 | 2 | Ns | - | 2/21 |

^*^1 misincorporation per 13,263 bp on the basis of RT-PCR amplification by PfuUltra. Ns=not significant
